# Anti-inflammatory and anti-nociceptive effects of Cinnamon and Clove essential oils nanogels: an in vivo study

**DOI:** 10.1186/s12906-022-03619-9

**Published:** 2022-05-20

**Authors:** Fariba Esmaeili, Masoumeh Zahmatkeshan, Yaser Yousefpoor, Hiva Alipanah, Ehsan Safari, Mahmoud Osanloo

**Affiliations:** 1 Department of Medical Nanotechnology, School of Advanced Technologies in Medicine, Tehran University of Medical Sciences, Tehran, Iran; 2 Cellular and Molecular Research Center, Iran University of Medical Sciences, Tehran, Iran; 3 Department of Medical Nanotechnology, Faculty of Advanced Technologies in Medicine, Iran University of Medical Sciences, Tehran, Iran; 4 Research Center of Advanced Technologies in Medicine, Torbat Heydariyeh University of Medical Sciences, Torbat Heydariyeh, Iran; 5 Department of Physiology, School of Medicine, Fasa University of Medical Sciences, Fasa, Iran; 6 Noncommunicable Disease Research Center, Fasa University of Medical Sciences, Fasa, Iran; 7 Department of Medical Nanotechnology, School of Advanced Technologies in Medicine, Fasa University of Medical Sciences, Fasa, Iran

**Keywords:** Analgesics, Nanomedicine, Painkiller, Paw edema test

## Abstract

**Background:**

Cinnamon (*Cinnamomum zeylanicum*) and Clove (*Syzygium aromaticum*) essential oils are two medicinally important plant-derived substances with a wide range of biological properties. Besides, nanoemulsion-based gels have been widely used to increase topical drug delivery and effectiveness.

**Methods:**

This study aimed to explore the anti-inflammatory effect (paw edema test) and the anti-nociceptive effect (hot plate and formalin test) of nanoemulsion-based gels containing the essential oils in the animal model. Cinnamon and Clove essential oils nanoemulsions with droplet sizes of 28 ± 6 nm and 12 ± 3 nm were first prepared. By adding carboxymethylcellulose (3.5% w/v), the nanoemulsions were then gelified. Finally, the nanogels were characterized by ATR-FTIR analysis and were used as topical pre-treatment before induction of inflammation or pain in acute and chronic analgesic experimental studies.

**Results:**

The paw edema and formalin findings showed that the nanogels formulations possess significant anti-nociceptive and anti-inflammatory effects.

**Conclusion:**

The prepared nanogels could be considered as analgesic drugs for inhibiting the inflammation and pain of diseases.

## Introduction

Inflammation, as a natural reaction at the site of external injurious (events) environments, plays a vital role in the pathogenesis of many chronic diseases such as rheumatoid arthritis, diabetes, and cancer [1, 2]. If the inflammation is severe at the tissue level, damage to nerves can cause pain signals that transmit through neurons to the brain [3, 4]. Nowadays, knowledge about pain and its mechanisms, especially neurophysiological and neuropathic pains, has increased significantly [5]. For example, emerging evidence suggests that inflammation and the release of inflammatory mediators from damaged tissues can cause pain [6]. Anti-inflammatory or painkiller nanodrugs drugs such as steroidal and non-steroidal are commonly used for inflammatory diseases and their related pains while having limited efficiency with side effects [7, 8]. Therefore, attempts to develop green nanodrugs as an important source of novel therapeutics have received more attention recently [9, 10].

Essential oils (EO)s as secondary metabolites of plants have recently been considered to treat inflammation and pain [11, 12]. For instance, Cinnamon EO (*Cinnamomum zeylanicum*), a spice derived from the inner bark of the genus Cinnamomum trees, has promising inflammatory effects [13, 14]. Some reports regarding Cinnamon’s anti-nociceptive and antipyretic effects in bronchitis, rheumatism, cold, fever, headache, and muscular pain [15, 16]. Also, the Cloves (*Syzygium aromaticum*) belongs to the *Myrtaceae* family, which is a nail-shaped dried flower bud [17, 18]; its EO has been traditionally applied in aromatherapy, relieving headaches, joint pain, toothaches, and oral antiseptic [19, 20]. Furthermore, Clove EO has also been used in dental emergencies as an asymptomatic reliever of toothache and anti-inflammatory in the mouth and throat [21, 22]. Also, other applications of Clove EO and Clove extracts have been reported, such as antimutagenic, antioxidant, antithrombotic, antiparasitic, antibacterial, antiviral, and antifungal activities [23, 24].

Formulating of EOs as nanoformulations is a promising approach for increasing their efficacy [25, 26]. Among the nanoformulations, nanoemulsions are more considered due to their relatively fewer side effects, bioavailability, and simpler preparation methods [27–29]. Nanoemulsions are biphasic transparent dispersions composed of oil and water phases stabilized by surfactants/co-surfactant. Such systems are stable droplets over aggregation or creaming processes because of their droplet size (less than 200 nm) [30, 31]. Nanoemulsions as effective topical delivery systems have demonstrated favorable characteristics, including enhanced permeability without skin irritations [32, 33]. However, transforming nanoemulsions to gel improves the topical administration and physical and thermal stability of EOs [34, 35].

To the best of our knowledge, the anti-nociceptive and anti-inflammatory effects of nanoemulsion-based gels of Cinnamon and Clove EOs have not been investigated. Therefore, their efficacy as a topical delivery system against inflammation and nociception was investigated in this study.

## Materials and methods

### Materials

λ-Carrageenan and carboxymethylcellulose (medium viscosity) were supplied from Sigma-Aldrich (Germany). Formalin (HCHO) and tween 20 were purchased from Merck Chemicals (Germany). Cinnamon and Clove EOs were bought from Green Plants of Life Co. and Zardband Pharmaceuticals Co. (Iran).

Twenty-four Wistar male rats weighing 180 ± 20 g were used. They were kept in the Standard Laboratory Animal Guidelines, and the experimental protocols were approved by the Tehran University of Medical Sciences Ethical Committee (code 91–01–87-17,072). Rats were randomly divided into 4 groups (*n* = 6). Each group was treated with 100 mg of the following samples, including distilled water as negative control (D.W.), blank gel, Cinnamon-nanogel (cinnamon- NG), and Clove-nanogel (clove-NG).

### Preparation and characteristics of nanoemulsions-based gels of Cinnamon and Clove EOs

For the preparation of nanoemulsions, at first, Cinnamon and Clove EOs (2.5% v/v) were added to tubes containing tween-20 (7.5 and 10% v/v, respectively), which were on stirrer equipment at 2000 rpm at room temperature (MS-300HS, Protraction Intertrade Co., Korea). Then, distilled water was added at room temperature dropwise to the desired volume, stirring at 2000 rpm for 40 minutes. Next, nanogels were prepared by adding carboxymethylcellulose (CMC 3.5% w/v) to the as-prepared nanoemulsions while mixing in a mild condition (180 rpm) for 4 h. The prepared gels were abbreviated as cinnamon-NG and clove-NG. Similarly, a blank gel was also prepared with distilled water.

The mean droplets size of nanoemulsions was measured by dynamic light scattering (DLS) at a scattering angle of 90^°^ using Scatteroscope (K-one Ltd. Korea). Droplet size distribution was calculated using the following equation, D90-D10/D50; D was the diameter of droplets, and D10, D50, and D90 were the percentile of droplets with a diameter lower than these values. The chemical spectra of nanoemulsions containing Cinnamon and Clove EOs, CMC powder, and nanogels were analyzed by Attenuated Total Reflection-Fourier-Transform InFrared (ATR-FTIR) using an infrared instrument in a wavenumber 400–4000 cm^− 1^ (Bruker, Tensor II, Germany) without any sample preparation process.

### Acute anti-nociceptive studies

The hot plate test was carried out to assess the acute anti-nociceptive activity of cinnamon-NG and clove-NG [36]. For this purpose, topical pre-treatment was carried out with 100 mg of the nanogels for each rubbing on the left hind paw for an hour with intervals of 15 minutes. For topical adsorption of nanogels, the rats were allowed 1 h after pre-treatment. Then rats were then placed on a hot plate (53 ± 2 °C). The response latency to the thermal stimulus was considered as the sign of nociception. The response latency was measured as the time taken by the animal for nocifensive behaviors, including paw licking, jumping, or flinching. The first nocifensive behaviors were recorded, and rats were immediately removed from the hot plate. To prevent tissue damage, a cut-off period was taken as the 60 s.

### Acute and chronic anti-nociceptive studies

Formalin test was used to assess gels’ acute and chronic anti-nociceptive effects [37]. After 2 weeks after the hot plate test, the rats were anesthetized, and topical pre-treatment was carried out with 100 mg of the nanogels (contains 2.5 mg of EOs) for each rubbing on the left hind paw for an hour with intervals of 15 minutes (totally 10 mg EO). For topical adsorption of nanogels, the rats were allowed 1 h after pre-treatment. Then acute pain was induced by subcutaneous injection of formalin 1% into the dorsal surface of the paw. Afterward, the rats were immediately placed into a flat plexiglass chamber (30 × 30 × 30 cm) with a mirror at 45^°^ angles for observed the animals’ response to pain-related behaviors during 15 s periods for 1 h at 1 min intervals based on the numerical scale in Table 1 (Dubuisson, Dennis) [38]. Following formalin injection, anti-nociceptive effects were assessed in two phases: in phase-1 acute pain was recorded 5 min after the injection, and in phase-2 chronic pain was recorded 15–60 min after the injection were assessed. The weighted scores or rating scale method (Eq. 1) was used to quantify the pain [39].

1 $$Average\ weighted\ score=\frac{0T0+1T1+2T2+3T3}{T0+T1+T2+T3}$$

**Table 1:** Categories of formalin-induced nociception behaviors in rats

Pain behavior scale	Pain score
Rat stands or walks firmly on injected paw	0
The injected paw was not fully lifted	1
The injected paw was wholly lifted off the floor	2
The rat licked or chewed the injected paw	3

The second number of animals in each category is defined as T and multiplied by its given weight score for 5 min test. The total time was 300 s, and the pain scores were generated with a range of 0–3.

In addition to anti-nociceptive effects, the total licking times of the injected paw were recorded in two phases; the first phase 0–5 min (acute) and the second phase 15–60 min (chronic), which represent both neurogenic and inflammatory pain responses, respectively.

### Anti-inflammatory studies

According to previous studies, the carrageenan-induced model performed the paw edema test to investigate the anti-inflammatory effects of the nanogels [9]. After deep anesthesia of rats, topical pre-treatment was carried out with 100 mg of the nanogels for each rubbing on the right hind paw for an hour with intervals of 15 minutes. For topical adsorption of nanogels, the rats were allowed 1 h after pre-treatment. Then inflammation was induced by injection of 0.1 mL freshly carrageenan solution (1% w/v in normal saline 0.9%) into the sub-plantar region. Then paw edema (average volume of paw swelling) was measured by using a digital caliper up to 5 h with 1 h intervals following carrageenan injection. Finally, the paw edema percentage was calculated by Eq. 2. Where Va indicates the paw diameters after injecting carrageenan and Vb denotes the paw diameters before injecting carrageenan

2 $$Paw\ edema\ \left(\%\right)=\frac{Va- Vb}{Vb}\times 100$$

### Statistical analysis

One-way analysis of variance and Tukey comparison were performed to assess the statistical significance of differences among groups. Results with a *p* value< 0.05 were considered statistically significant. Statistical analyses were carried out using the SPSS software, v. 21 (SPSS, Inc., USA).

## Results

### Determining the size of the nanoemulsions

**Fig. 1:**
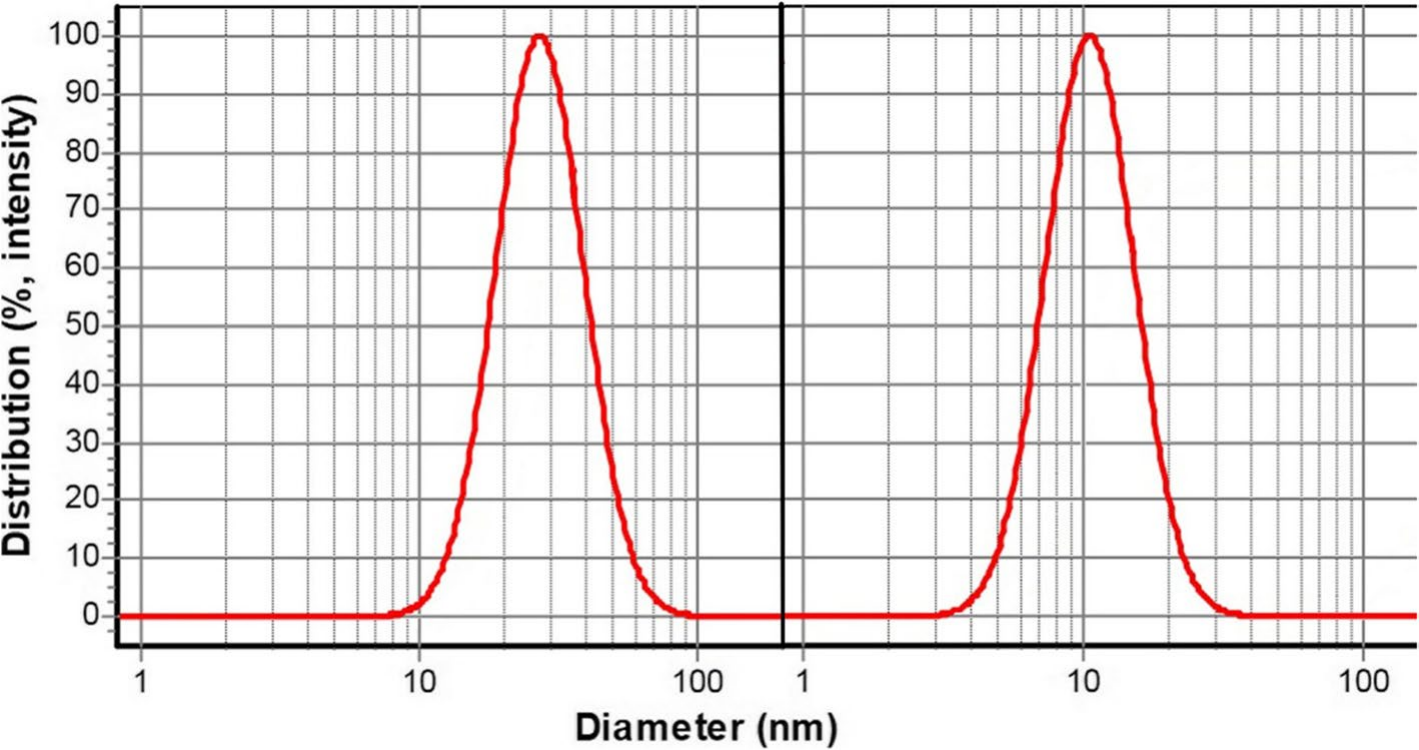
Left curve: Cinnamon EO nanoemulsion with mean droplet size ± SD: 28 ± 6 nm; Right curve: Clove EO nanoemulsion with droplet size ± SD: 12 ± 3 nm

Figure 1 shows the mean droplets size and size distribution of nanoemulsions containing Cinnamon and Clove EOs, which had a size of less than 100 nm and droplet size distribution of less than 1.

### Chemical properties of the nanogels

**Fig. 2:**
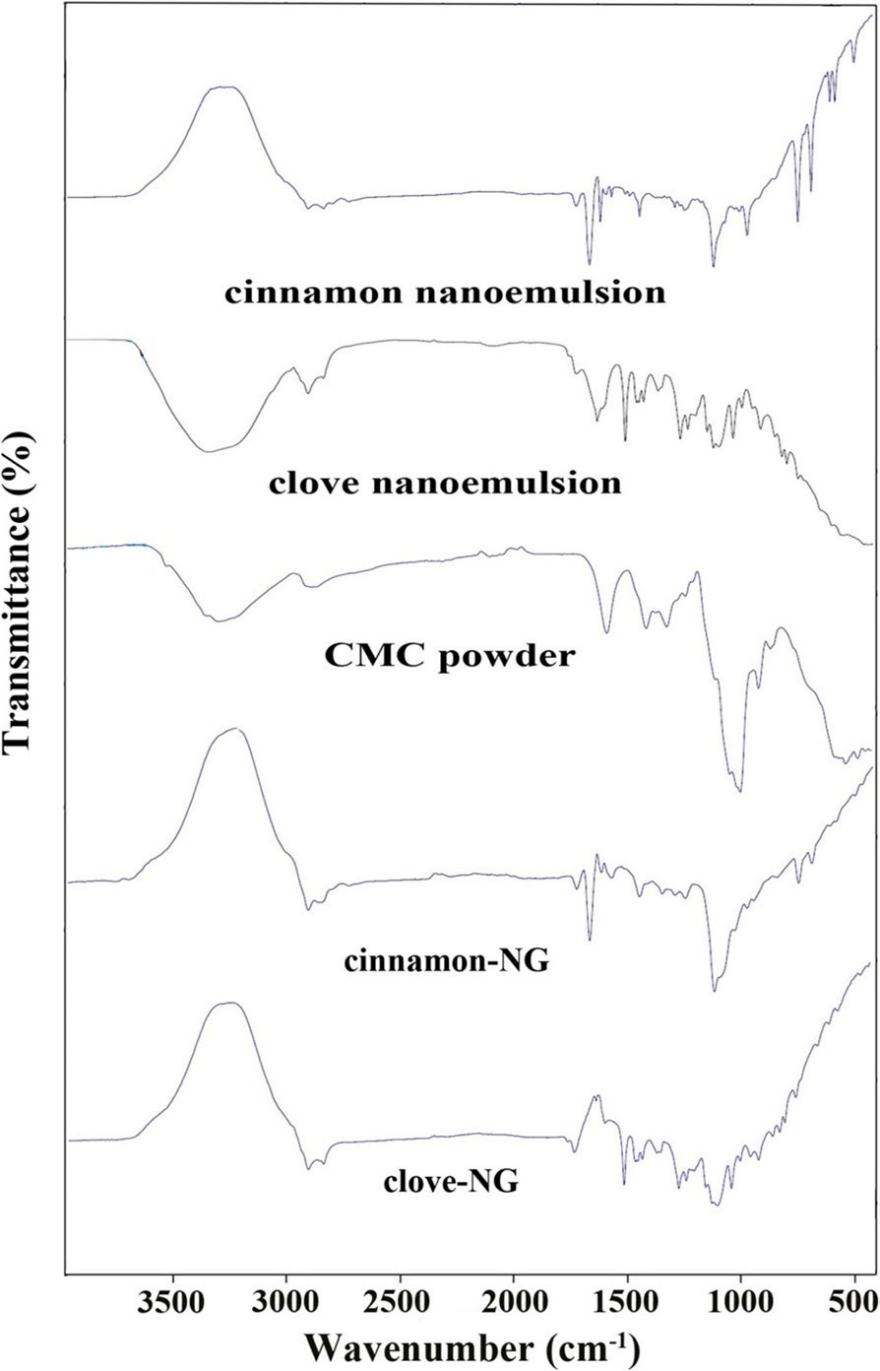
ATR-FTIR spectra of the samples (CMC: carboxymethylcellulose, NG: nanogel)

Figure 2 shows the ATR-FTIR spectra of the samples in terms of transmittance rate (%). In the Cinnamon nanoemulsions spectrum, the peak at 2922 and 2854 cm^− 1^ are attributed to C-H stretching. The peak at 1732 cm^− 1^ exhibited C=O stretching, and the characteristic absorption at around 1450 cm^− 1^ shows CH_2_ bending. In the Clove nanoemulsions spectrum, the broadband at 3358 cm^− 1^ is attributed to O-H stretching vibration due to hydrogen bonding between Clove EO and tween 20 molecules. The peak at 2924 cm^− 1^ is attributed to C-H stretching, and the peak at 1731 and 1631 cm^− 1^ exhibited C=O stretching. The characteristic absorption at around 1463 cm^− 1^ showed CH_2_ bending, and the peak at 1003 cm^− 1^ is attributed to C-O stretching.

ATR-FTIR spectrum of CMC showed the broad bands at 3321 cm^− 1^ can be attributed to the stretching of the hydroxyl group O-H due to H-bonding, the strong band at 1589 cm^− 1^ related to COO- group (asymmetric stretching), and 1413 cm^− 1^ related to COO- (symmetric stretching). The characteristic absorption at around 993 cm^− 1^ is attributed to C-O stretching. Cinnamon-NG spectrum peak at about 2922 cm^− 1^ is attributed to C-H stretching due to EO, tween, and CMC. The peak at 1731 and 1674 cm^− 1^ exhibited C=O stretching, representing the carbonyl group in Cinnamon EO with tween molecules. The sharp and strong peak at 1120 cm^− 1^ is attributed to C-O stretching. Noticeably, the COO^−^ band at 1589 cm^− 1^ in the presence of CMC was shifted toward the lower wavenumber at 1576 cm^− 1^, confirming the association of CMC with tween through intermolecular H-bonding.

Moreover, in the spectrum of clove-NG, the peak at 2922 and 2854 cm^− 1^ are attributed to C-H stretching due to Clove EO, tween, and CMC. The peak at 1735 cm^− 1^ exhibited C=O stretching, representing the overlap carbonyl group in Clove EO with tween molecules. The sharp and strong peak at 1097 cm^− 1^ is attributed to C-O stretching. Noticeably, the COO^−^ band at 1589 cm^− 1^ in the presence of CMC was shifted toward a lower wavenumber at 1513 cm^− 1^, confirming the association of CMC with tween through intermolecular H-bonding.

### Acute anti-nociceptive studies

**Fig. 3:**
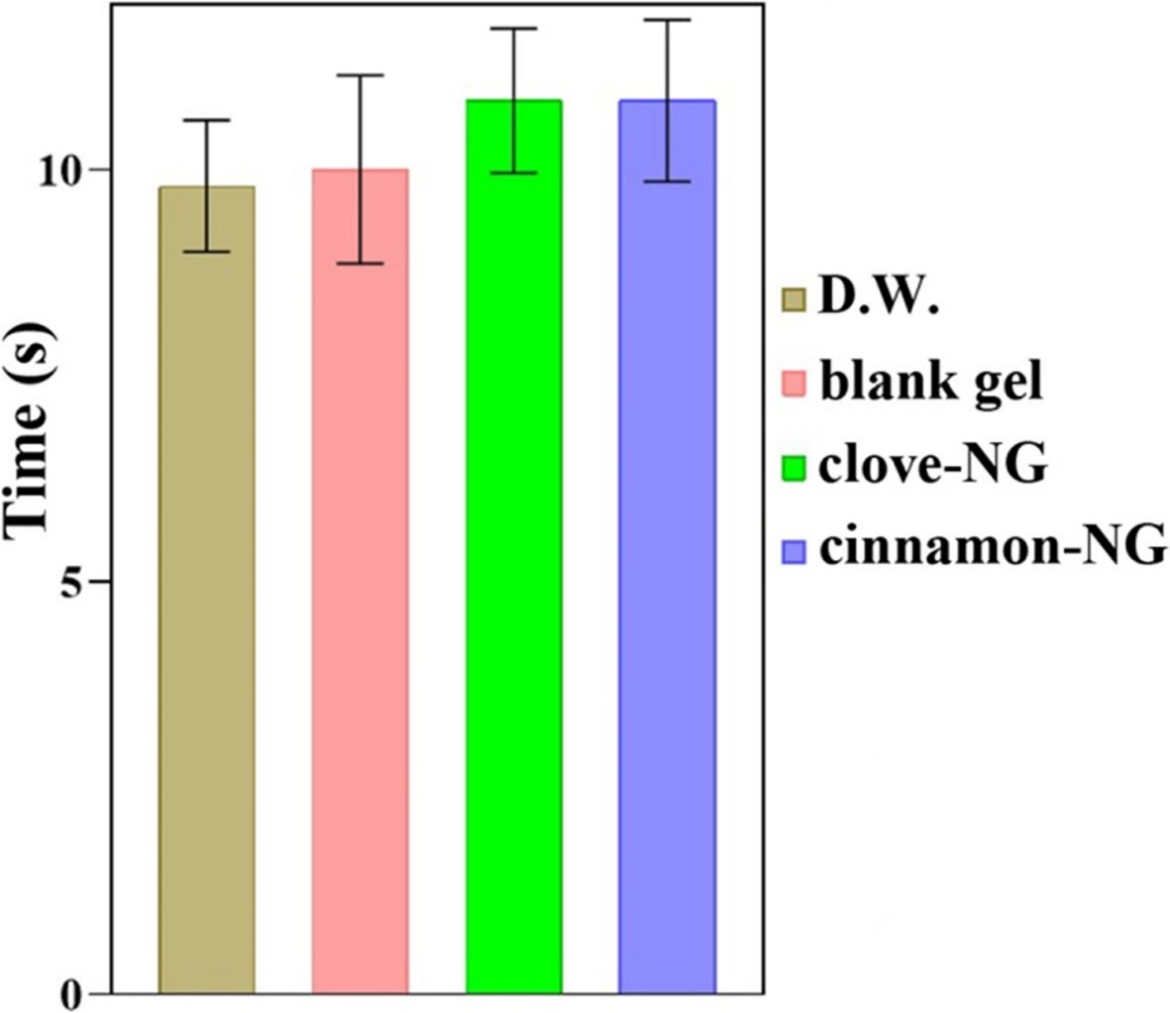
The acute anti-nociceptive activity of cinnamon-NG and clove-NG on reaction latency in hot plate test; no significant differences between samples. Investigated groups (*n* = 6) include distilled water (D.W.: control group), blank gel, cinnamon-NG, and clove-NG. The data were presented as mean ± SE

Figure 3 shows the experimental results of the hot plate test, the thermal anti-nociceptive activity of formulations, and the central pain index. As the details direct, topical administration of cinnamon-NG and clove-NG failed to prolong latency time to the hot plate painful stimulus compared to the control group.

### Acute and chronic anti-nociceptive studies

**Fig. 4:**
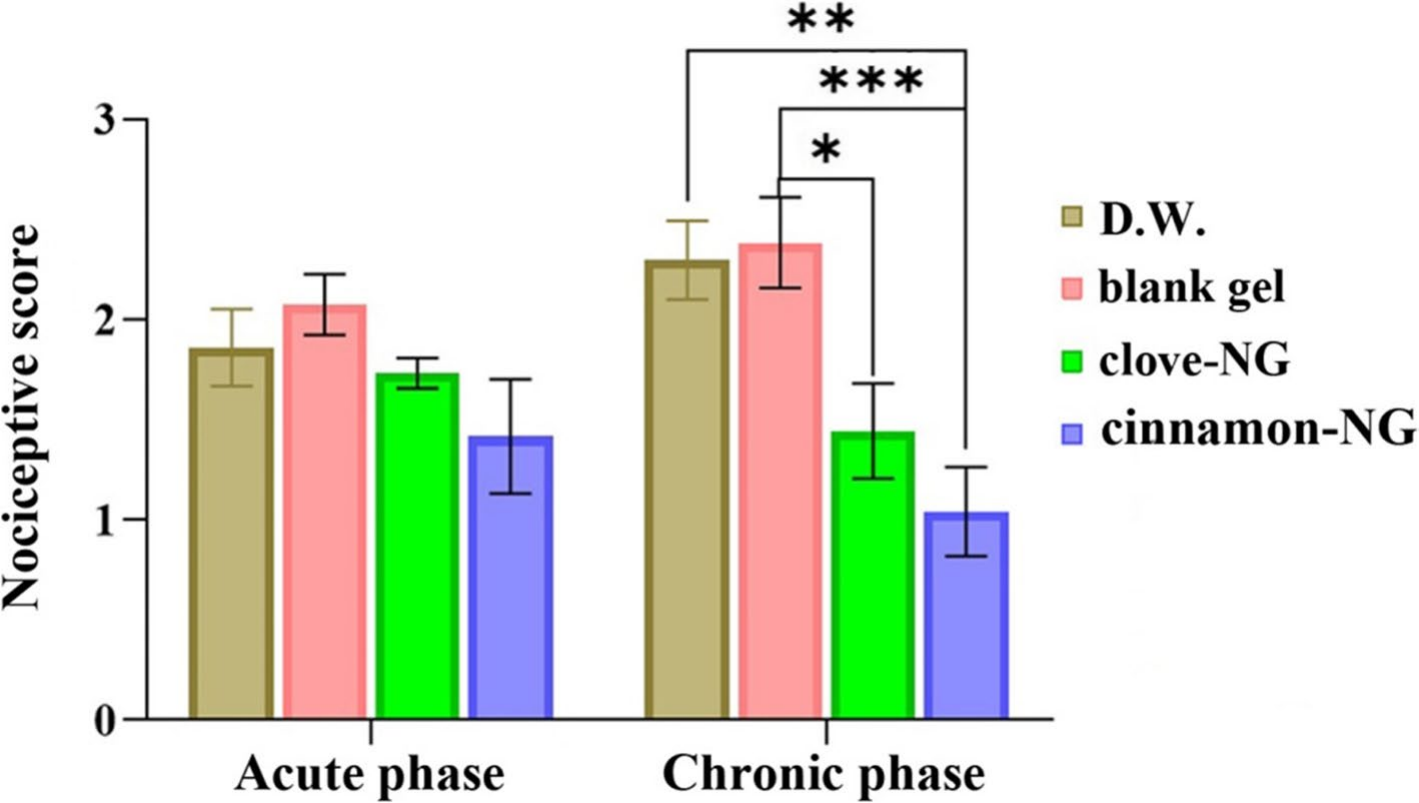
Comparison of acute and chronic phases results of formalin test after treatment with distilled water (D.W.: control group), cinnamon-NG, and clove-NG. Each value represents mean ± SEM, *P < 0.05, **P < 0.01, ***P < 0.001

Formalin test was used to assess acute and chronic anti-nociceptive responses. The anti-nociceptive results of formulations through chemical stimuli of formalin were reported in Fig. 4 and Table 2. Noteworthy, no anesthesia, movement impairments, and respiratory insufficiency were seen in the animals during the test. In the second phase of the formalin test, pre-treatment rats with cinnamon-NG and clove-NG show significant anti-nociceptive activity and reduced nociceptive scores. However, this effect was more significant and noticeable in cinnamon-NG. In detail, as the graph shows, no significant difference was observed between groups up to 20 min following formalin injection. However, in the groups pre-treated with cinnamon-NG and clove-NG, an immediate reduction in nociceptive scores was observed at 25 min.

**Table 2:** Statistical comparison between the groups on formalin test (chronic phase) including distilled water (D.W.: control group), cinnamon-NG, and clove-NG

Time (min)	Groups	***P-***Value
25	D.W. vs. cinnamon-NG	0.0194
	blank gel vs. clove-NG	0.0072
	blank gel vs. cinnamon-NG	0.0017
30	D.W. vs. cinnamon-NG	0.0019
	blank gel vs. cinnamon-NG	0.0009
35	D.W. vs. cinnamon-NG	0.0099
	blank gel vs. clove-NG	0.0331
	blank gel vs. cinnamon-NG	0.0012
40	D.W. vs. cinnamon-NG	0.0005
	blank gel vs. cinnamon-NG	0.0003
45	D.W. vs. cinnamon-NG	0.0003
	blank gel vs. cinnamon-NG	0.0002
	clove-NG vs. cinnamon-NG	0.0241
50	D.W. vs. clove-NG	0.0007
	D.W. vs. cinnamon-NG	0.0006
	blank gel vs. clove-NG	0.0037
	blank gel vs. cinnamon-NG	0.0036
55	D.W. vs. clove-NG	0.0418
	D.W. vs. cinnamon-NG	0.0007
	blank gel vs. clove-NG	0.0135
	blank gel vs. cinnamon-NG	0.0001
60	D.W. vs. clove-NG	0.0002
	D.W. vs. cinnamon-NG	0.0001
	blank gel vs. clove-NG	< 0.0001
	blank gel vs. cinnamon-NG	< 0.0001

Moreover, these groups remained lower than the blank gel and control groups (D.W.) until the end of the test. Worth mentioning that the significant differences in nociceptive scores between clove-NG and control were seen from 50 min onwards, while the significant ones between clove-NG and gel were observed at 25, 35, 50, 55, and 60 min. Meanwhile, the significant anti-nociceptive effect in the cinnamon-NG group compared with the control and gel groups was noticed at all-time points after 20 min, indicating a strong anti-nociceptive effect.

**Fig. 5:**
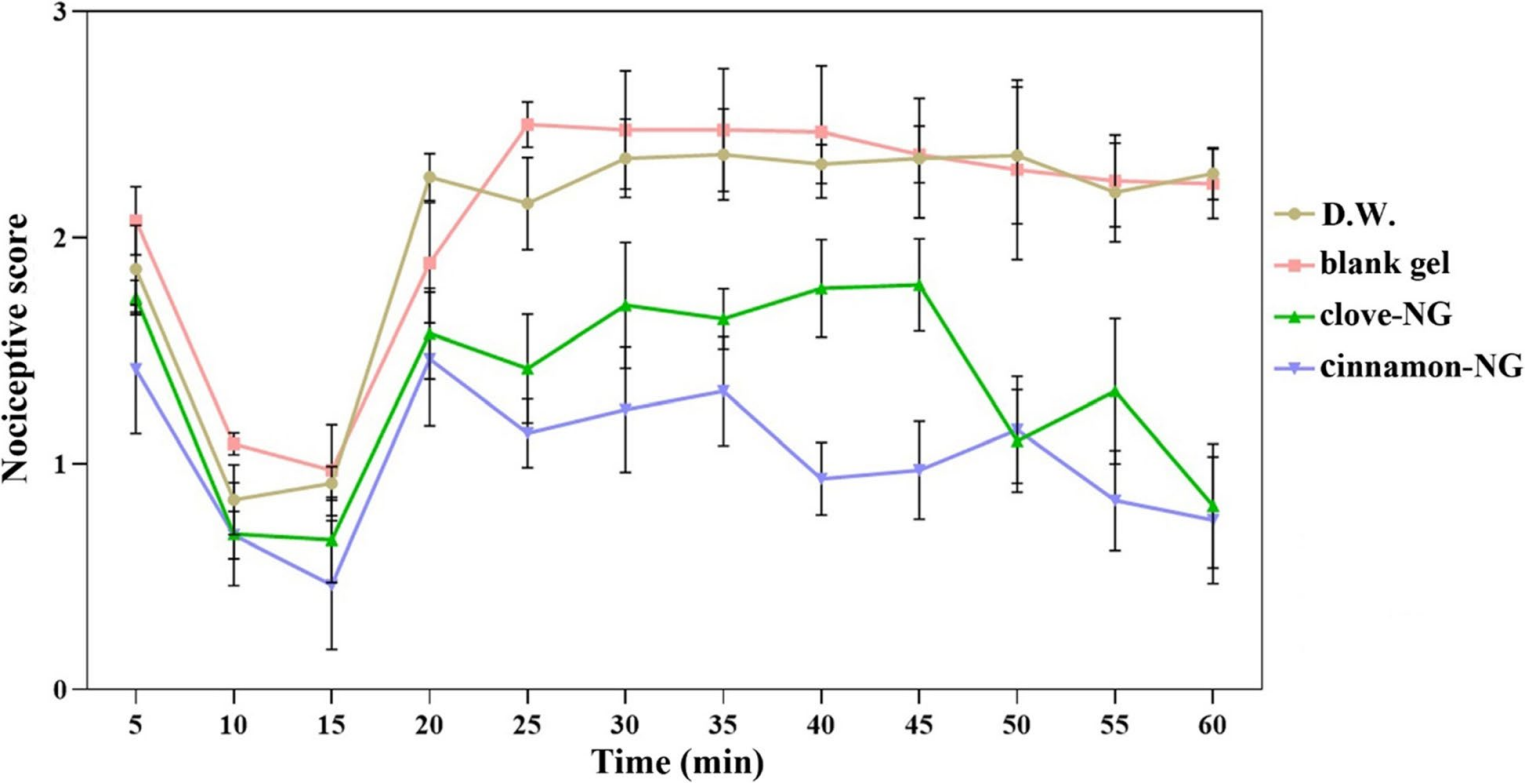
The effect of cinnamon-NG and clove-NG on rat nociceptive behavior. Groups (*n* = 6) include distilled water (D.W.: control group), cinnamon-NG, and clove-NG. Each value represents mean ± SE

The pain response of formulations to both acute and chronic phases of the formalin test is illustrated in Fig. 5. Our results indicated no significant difference between the tested group in the acute phase, while chronic pain intensity was significantly decreased in the cinnamon-NG and clove-NG groups. These findings are consistent with the results in Fig. 4. Therefore, both cinnamon-NG and clove-NG significantly affect the second phase of the nociceptive response in the formalin test and not in the first phase.

**Fig. 6:**
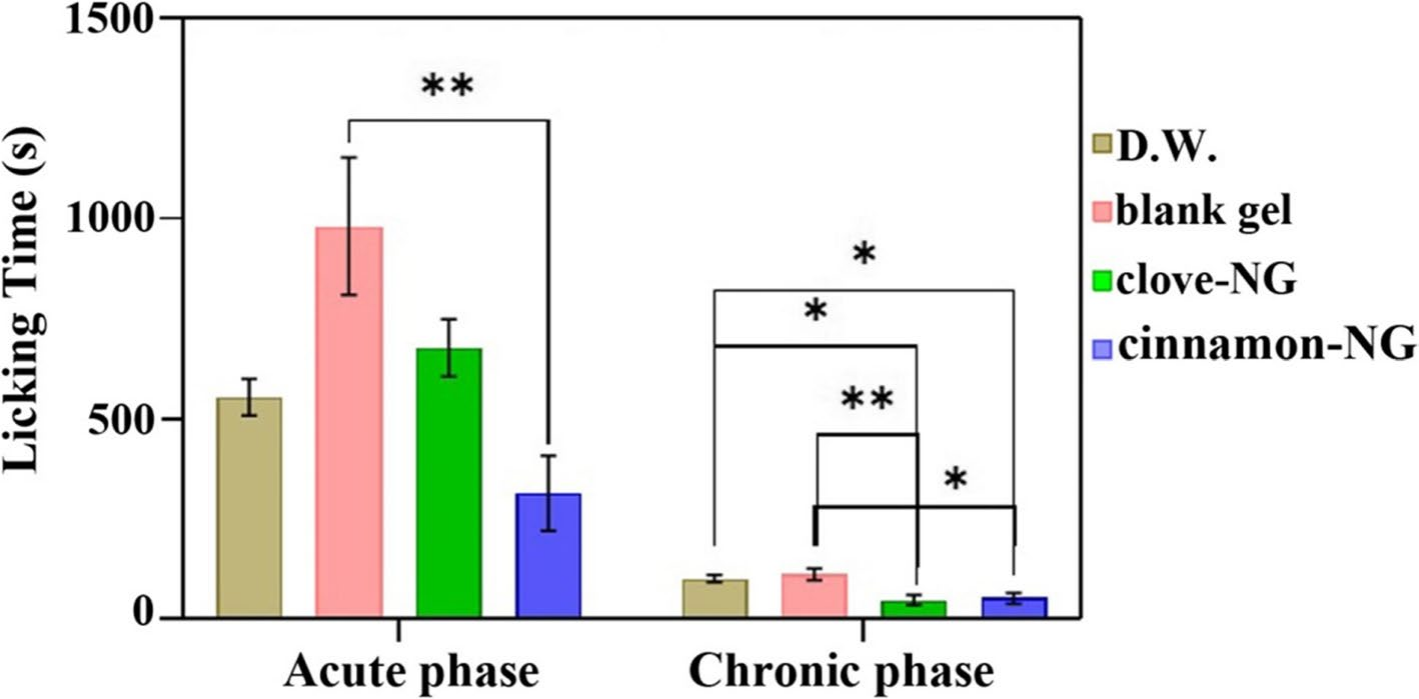
Comparison of licking time in acute and chronic phases of the formalin test after treatment with distilled water (D.W.: control group), blank gel, clove-NG, and cinnamon-NG. Each value represents mean ± SEM, *P < 0.05, **P < 0.01

As shown in Table 3, the paw licking time revealed a significant (*P* < 0.05) reduction in nociceptive response between different treated groups compared to controls. The cinnamon-NG and clove-NG suppressed licking time more effectively in the late phase. However, cinnamon-NG also shortened licking time in the early phase (Fig. 6).

**Table 3:** Statistical comparison of the groups on formalin-induced paw licking (in seconds) in acute and chronic phases, including distilled water (D.W.: control group), blank gel, cinnamon-NG, and clove-NG

Phase	Groups	***P***-value
**Acute**	blank gel vs. cinnamon-NG	0.0031
**Chronic**	D.W. vs. clove-NG	0.0205
	D.W. vs. cinnamon-NG	0.0483
	blank gel vs. clove-NG	0.0091
	blank gel vs. cinnamon-NG	0.0213

### Anti-inflammatory studies

**Fig. 7:**
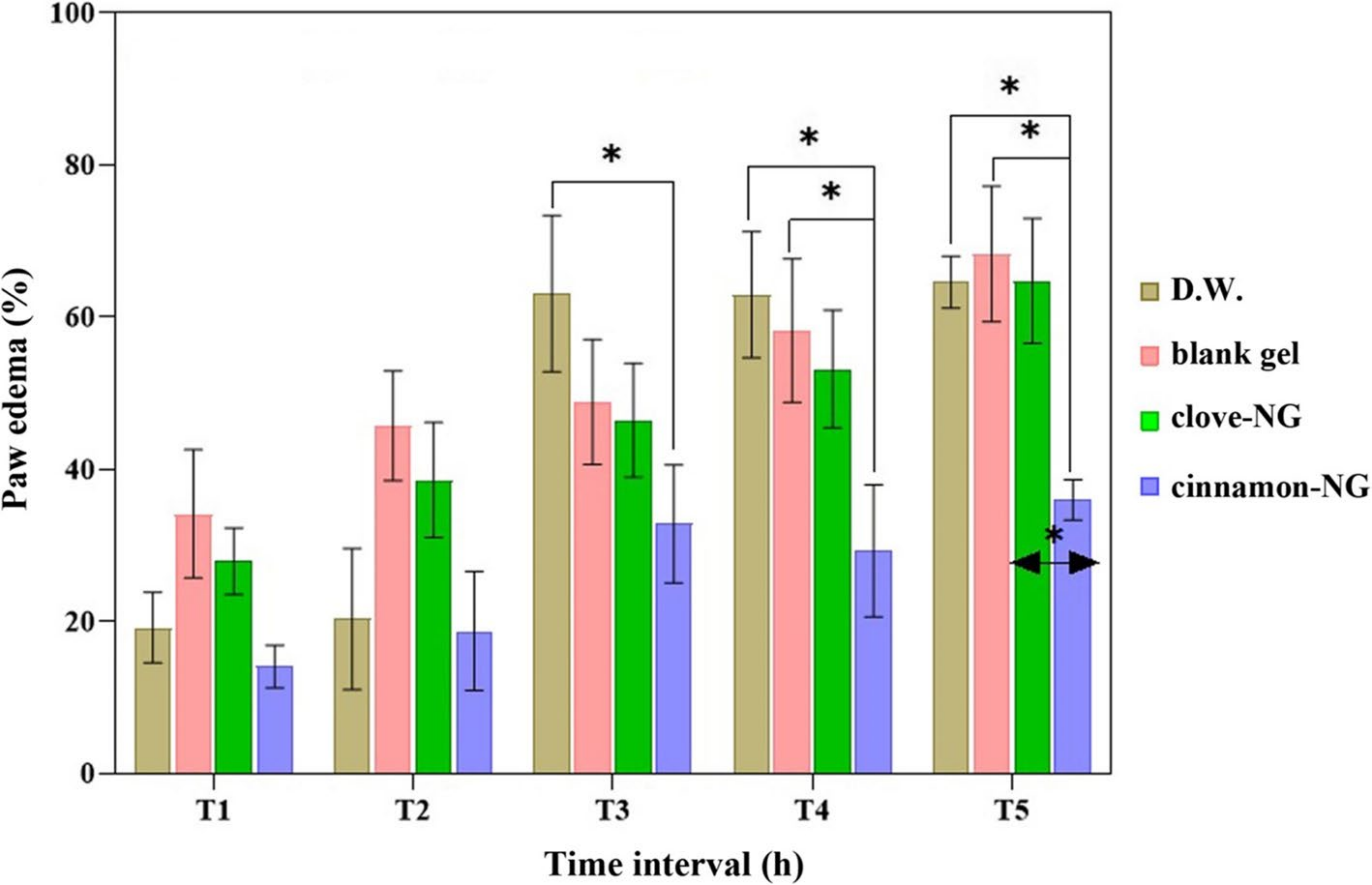
The effect of cinnamon-NG and clove-NG in a rat paw edema induced by carrageenan model. Rats (*n* = 6) were pre-treated with distilled water (D.W.: control group), blank gel, cinnamon-NG, and clove-NG. The measurements were obtained at 1, 2, 3, 4, and 5 hours after the carrageenan sub-plantar injection (1%, 100 μl). *P < 0.05

The inhibition of carrageenan-induced paw edema in rats is a model for establishing the efficacy of anti-inflammatory drugs [40]. As shown in Fig. 7, cinnamon-NG suppressed carrageenan-induced rat paw edema more than other groups, including clove-NG, control, and blank gel groups. A maximum inhibitory effect was seen in the cinnamon-NG group 4 h after treatment. The clove-NG inhibitory effect was more than D.W. and blank gel groups at the 1st and 2nd h of the experiment; however, the differences were insignificant. On the other hand, compared to D.W., the cinnamon-NG group was effective against carrageenan-induced inflammation, especially at the 4th and 5th h of the test, suggesting longer biological life cinnamon-NG compared to the carrageenan with the most activity at the 3rd h [41].

## Discussions

In the current study, nanoemulsions containing cinnamon and clove EOs were prepared; as obtained droplet sizes were lower than 200 nm, their nanoscale sizes were confirmed. Moreover, a droplet size distribution of less than 1 confirms narrow size distribution and single sharp peaks referred to as monodisperse systems [26]. The low-Energy or spontaneous method is common for preparing EO-based nanoemulsions and preventing evaporating volatile compounds in EOs [42, 43]. This method involves mixing the components of nanoemulsions at room temperature without any external energies as a high-homogenizer or ultrasound [33].

Ingredients of the used EOs in the current study (i.e., Cinnamon and Clove) were identified using Gas Chromatography-Mass Spectrometry and reported in our previous reports. Cinnamaldehyde, with 62.04%, is the major component of Cinnamon EO, and linalool (6.96%), *trans*-caryophyllene (6.60%), *trans*-cinnamyl acetate (4.29%), and benzyl benzoate (3.32%) are other major constituents [44]. Besides, eugenol, with 65.41%, is the major component in Clove EO. *trans*-caryophyllene (12.06%), eugenol acetate (9.85%), caryophyllene oxide (3.00%), and α-humulene (1.73%) are other major constituents [45].

Pro-inflammatory agents, including carrageenan, can affect vascular permeability and blood cells migration through modulated nitric oxide (NO) secretion [46, 47]. On the other hand, the production of pro-inflammatory mediators is stimulated by inducing nitric synthase (iNOS) and cyclooxygenase-2 (COX-2) (50, 51). It has been reported that eugenol and cinnamaldehyde, the main component of Clove EO and Cinnamon EO, demonstrated anti-inflammatory effects similar to COX inhibitors, including indomethacin and celecoxib [17, 48]. In addition, cinnamaldehyde inhibits lipopolysaccharide-induced chondrocyte inflammation [49]. Besides, eugenol in LPS-stimulated mouse macrophages showed a COX-2 inhibitory effect [50, 51]. This result suggests that cinnamon-NG and clove-NG could be candidates for further developing anti-inflammatory drugs.

It has been proposed that the analgesic effect of EO of Clove and Cinnamon may be due to these main ingredients (eugenol and cinnamaldehyde) [52, 53]. Their administration as analgesic agents in experimental models of pain in mice was reported [54, 55]. Moreover, it has been reported that eugenol demonstrated a significant anti-nociceptive effect against chemical stimuli [56]. Therefore, it is proposed that eugenol predominantly prevents the peripheral pain mechanism. Two cinnamon-NG and clove-NG block the peripheral pain mechanism and cannot affect central pain through the hot plate test. The formalin (chemical stimuli) results are also inconsistent with these findings.

It is important to note that acute and chronic phases results of the formalin test showed that both cinnamon-NG and clove-NG significantly affect the second phase of the nociceptive response in the formalin test and not in the first phase. Similar observations have also been reported about other nanoemulsions [57]. In addition, it was found that topical application of Clove EO and intraperitoneal administration of Cinnamon extract significantly decreased acute and chronic pain in formalin tests [58, 59].

According to rat’s paw licking time results, cinnamon-NG inhibited the formalin-induced pain response in both phases, indicating the involvement of both peripheral and central mediated mechanisms. In one study, a significant reduction in pain response was found in *Cinnamomum zeylanicum* (200 and 400 mg/kg) treated groups during the first phase of the formalin test. However, during the second phase, a significant reduction in formalin-induced pain response was observed in 100, 200, and 400 mg/kg *C. zeylanicum* extract-treated groups compared to the control group [60]. Moreover, cinnamic alcohols (a phenylpropanoid of cinnamon)-treated animals (6.25, 12.5, 25 mg/kg) exhibited reduced paw licking behavior in the first and second phases of the formalin test. Cinnamic alcohol’s anxiolytic and antinociceptive-like effects were suggested to be due to GABAergic system modulation [61]. The clove-NG inhibited the rat’s paw licking time in the late phase. According to the literature, eugenol exhibited an antinociceptive effect more in the inflammatory phase than in the neurogenic phase in a formalin-induced licking pain model [56]. Clove oil also reduces pain response through a mainly peripheral action, as demonstrated by the formalin test and the tail-flick test, which indicated the participation of opioid receptors [62].

## Conclusion

This study developed cinnamon-NG and clove-NG as a topical delivery system. Our work has led us to conclude that nanoemulsion-based gel formulations, especially cinnamon-NG, could apply as anti-nociceptive and anti-inflammatory agents or promising therapeutics in relieving diseases accompanied by inflammation and pain.

## Data Availability

The authors confirm that the data supporting the findings of this study are available within the article and its supplementary materials. Raw data are available from FE upon a reasonable request.
